# Stromatolites on the rise in peat-bound karstic wetlands

**DOI:** 10.1038/s41598-017-15507-1

**Published:** 2017-11-13

**Authors:** Bernadette C. Proemse, Rolan S. Eberhard, Chris Sharples, John P. Bowman, Karen Richards, Michael Comfort, Leon A. Barmuta

**Affiliations:** 1 School of Biological Sciences, University of Tasmania, Private Bag 55, Hobart, Tasmania 7001 Australia; 2 Australian Centre for Research on Separation Science, University of Tasmania, Tasmania, 7001 Australia; 3 Department of Primary Industries, Parks, Water & Environment, GPO Box 44, Hobart, Tasmania 7001 Australia; 4 Geography and Spatial Science, University of Tasmania, Private Bag 76, Hobart, Tasmania 7001 Australia; 5 Tasmanian Institute of Agriculture, University of Tasmania, Private Bag 98, Hobart, Tasmania 7001 Australia

## Abstract

Stromatolites are the oldest evidence for life on Earth, but modern living examples are rare and predominantly occur in shallow marine or (hyper-) saline lacustrine environments, subject to exotic physico-chemical conditions. Here we report the discovery of living freshwater stromatolites in cool-temperate karstic wetlands in the Giblin River catchment of the UNESCO-listed Tasmanian Wilderness World Heritage Area, Australia. These stromatolites colonize the slopes of karstic spring mounds which create mildly alkaline (pH of 7.0-7.9) enclaves within an otherwise uniformly acidic organosol terrain. The freshwater emerging from the springs is Ca-HCO_3_ dominated and water temperatures show no evidence of geothermal heating. Using 16 S rRNA gene clone library analysis we revealed that the bacterial community is dominated by Cyanobacteria, Alphaproteobacteria and an unusually high proportion of Chloroflexi, followed by Armatimonadetes and Planctomycetes, and is therefore unique compared to other living examples. Macroinvertebrates are sparse and snails in particular are disadvantaged by the development of debilitating accumulations of carbonate on their shells, corroborating evidence that stromatolites flourish under conditions where predation by metazoans is suppressed. Our findings constitute a novel habitat for stromatolites because cool-temperate freshwater wetlands are not a conventional stromatolite niche, suggesting that stromatolites may be more common than previously thought.

## Introduction

Stromatolites are a form of microbialite with repetitive, laminated structures of biologically (typically cyanobacteria) mediated mineral precipitation^1,2^. These microbial accretions are the oldest evidence for life on Earth^2–6^ and debates continue on whether they evolved first on land or in the ocean^4,7–10^. Fossilized stromatolites and similar accretionary microbial mats have provided intriguing microbial archives for over a century^11,12^, revealing that microbialites were abundant in the shallow late-Archean and Proterozoic oceans, but declined with the emergence of multicellular life in the Cambrian^2,13^. The evolution of grazing metazoans has therefore been suggested as a primary cause of stromatolite decline during the history of life on Earth. However, the role of metazoans in limiting stromatolite formation has been questioned^14,15^, and a living example for the co-occurrence of stromatolites and benthic macroinvertebrates has recently been reported^16^. Evidence from gene sequencing also suggests that microbialites support diverse and distinct active eukaryotic communities which may influence microbialite structure^17^.

Modern living stromatolites are rare but occur in diverse habitats which are often subject to extreme conditions inhospitable to other life forms^1,12,18,19^. Well-known living examples are shallow marine stromatolites in Hamelin Pool, Shark Bay, Western Australia^2–6^, and the shallow subtidal stromatolites in Highborne Cay, Bahamas^4,7–10^. Other occurrences include (hyper-) saline lacustrine environments such as Storr’s Lake, Bahamas^11,12^, a hyper-saline lake of the Kiritimati Atoll, Central Pacific^2,20^, high-altitude Lake Socompa, Argentina^14,15^, and supratidal pools along the coastline of South Africa^16,21^. However, stromatolites growing in low-salinity, low-temperature freshwaters have also been recognized at localities such as: Ruidera Pools Natural Park, Spain^22^, Pavilion Lake, British Columbia, Canada^23^, karst-water creeks in Germany and France^24,25^, cenote lakes in south-eastern mainland Australia^26^, and tufa depositing streams in SW Japan^27^.

**Figure 1:**
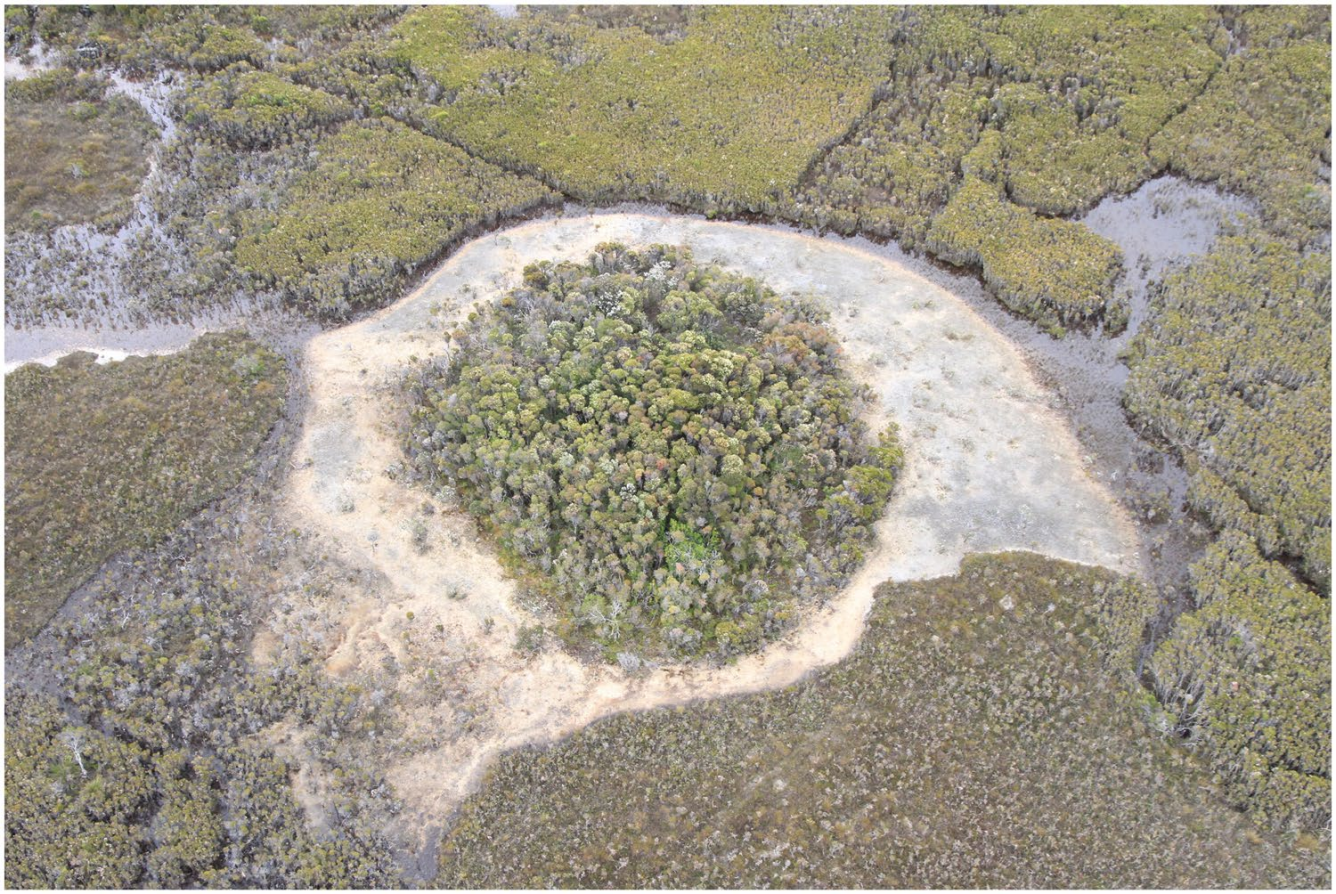
Aerial view of a prominent Giblin River spring mound (site GR5 and transect). The shrubby centre conceals marshy ground and shallow ponds which discharge groundwater. The pale outer band is calcareous mud and tufa colonized by stromatolites. This example is 60 m in diameter.

The Giblin River catchment and certain others in south-west Tasmania, Australia, contain significant concentrations of unusual wetlands comprising poorly drained, sparsely vegetated sandy to gravelly flats of variable size and shape (Fig. 1, SI Fig. 1). Older literature refer to these features as ‘alkaline pans’^28^, because the water is neutral to mildly alkaline (pH ~7–8), creating exceptional pH gradients across the boundary between the wetlands and surrounding acidic blanket bogs (pH ~4–5). This is striking because the majority of surface waters in south-west Tasmania are strongly acidic due to the high humic content of the soil^29^. We refer to these features as ‘peat-bound karstic wetlands’, because they comprise ‘islands’ of peat-free ground within otherwise monotonous organosol terrain of karstic limestone and dolomite valleys (the term alkaline pan is conventionally reserved for dryland evaporite features). Previously interpreted as ephemeral disturbance features due to burning of the peat with resultant exposure of underlying alkaline substrates, water chemistry data lead us to re-interpret the peat-bound karstic wetlands as spring-fed groundwater-dependent ecosystems.

**Figure 2:**
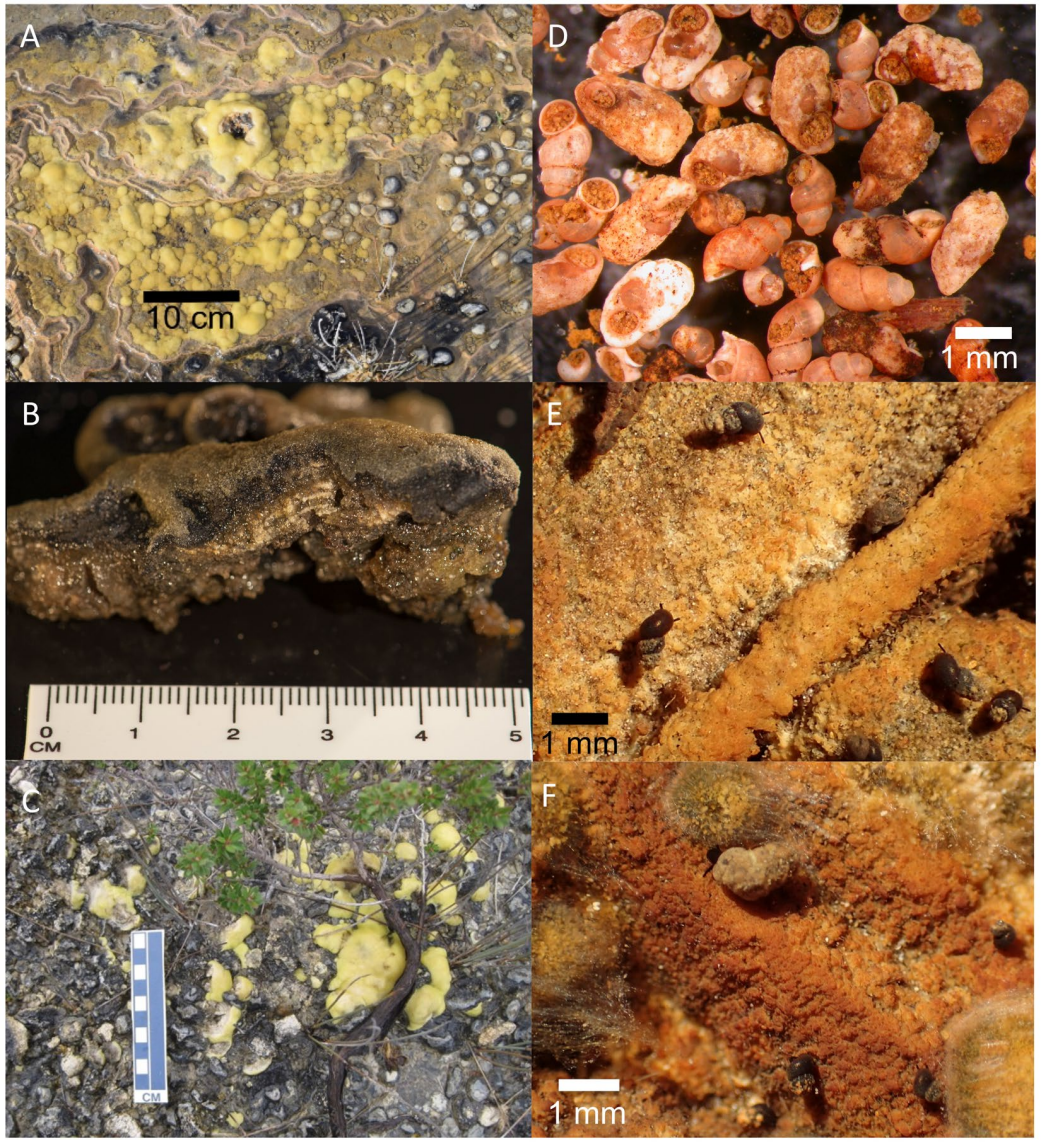
Giblin River stromatolites and macroinvertebrates: stromatlites in growth position (**A,C**); sectioned sample with calcite laminations (**B**), photograph taken by R. Wiltshire; dead and calcified snail shells from mound slopes (**D**) and live grazing snails (*Austropyrgus pisinnus*.) with light (**E**) and heavy (**F**) Ca-encrustations.

Recent visits to peat-bound karstic wetlands in the Giblin River catchment revealed that their surfaces are colonized by aggregations of stromatolites (Fig. 2A). There are no previous records of stromatolites in Tasmania, except as Ordovician and Precambrian fossils^30^. We have studied the microbial structure of the Giblin River stromatolites and characterize the physico-chemical conditions of their habitat to determine environmental parameters that control and allow modern freshwater stromatolite formation in this region.

## Results and Discussion

Stromatolites colonize the lower, peripheral slopes of the Giblin River spring mounds, forming smooth mats of yellowish and greenish, globular structures growing on the wetted surface of tufa barriers (Fig. 2A,B and C). The stromatolites are not fully submerged in water and almost appear as a ‘terrestrial’ variant rising several centimeters above the wet surface (Fig. 2C). The high amount of precipitation in this area (~2500 mm) may therefore contribute to stromatolite survival at this site. The largest examples are ~10 cm in diameter but most are considerably smaller. Internally, the structure of the stromatolites presents a regular layered succession of pale and dark laminae, each about a millimeter thick in cross section (Fig. 2B). Raman spectroscopy of the pale layers showed distinct peaks at around 288, 719 and 1090 cm^−1^ (SI Fig. 2), revealing that they are crystalline calcite (CaCO_3_).

### Water Chemistry

**Figure 3:**
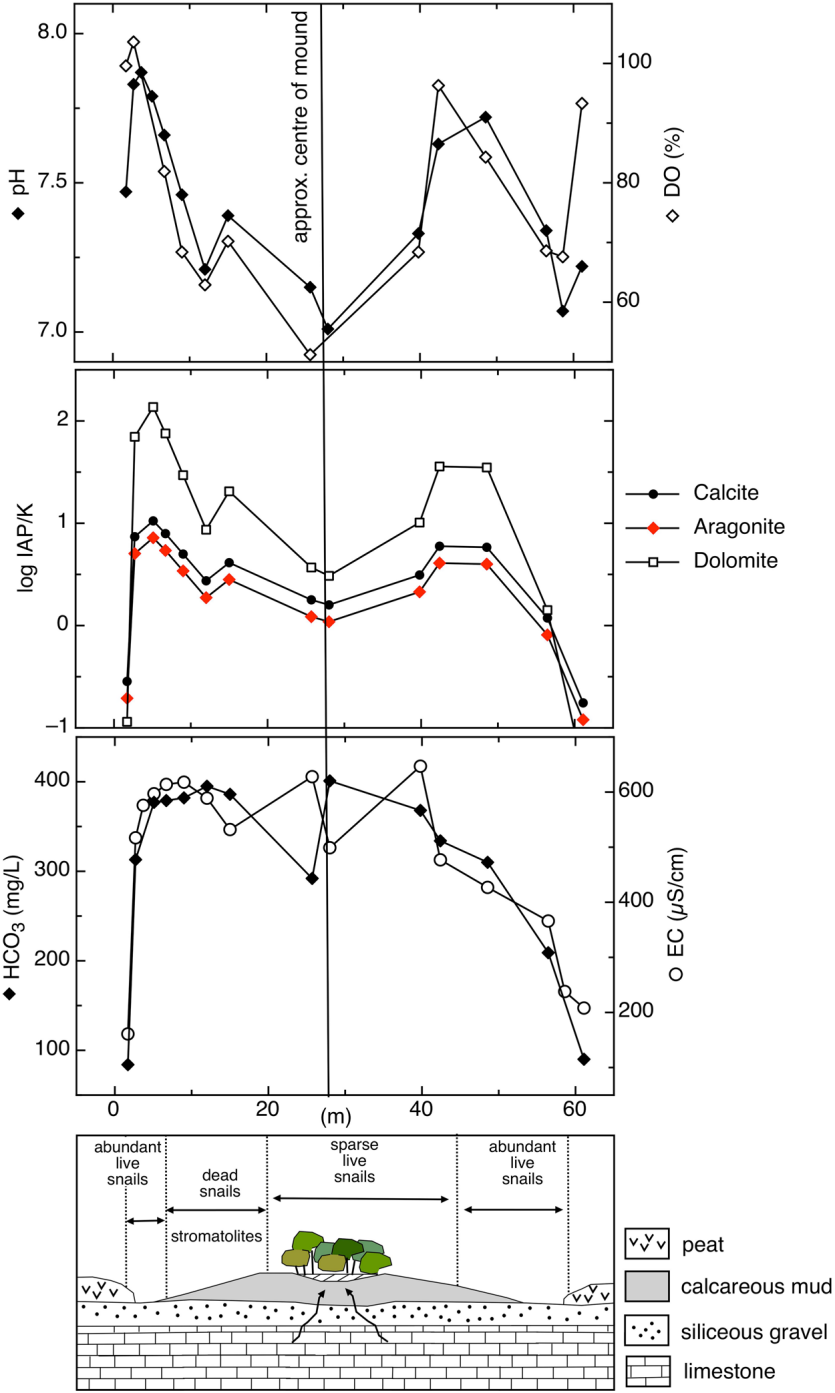
pH, dissolved oxygen (DO), saturation indices (logIAP/K), bicarbonate (HCO_3_) and electrical conductivity (EC) results along transect across the mound. Concentrations of dead Ca-encrusted snails on the middle slopes are associated with a shift towards higher pH and SI, as shown in the lower schematic. In fact, most parts of the mound, except the central pond and mixing zone on the periphery, show evidence of active calcite deposition.

Water chemistry data corroborates our interpretation of the Giblin River wetland as a class of groundwater-dependent ecosystem. Water from the spring mounds (GR2, GR5)  is mildly alkaline freshwater with a pH of 7.5–7.6, zero salinity, and electrical conductivity of 618–640 µS cm^−1^. The temperature indicates negligible geothermal heating (10.6–14.4 °C). These waters are Ca-HCO_3_ dominated, followed by Cl, Mg, Na and SO_4_ (SI Fig. 3, SI Table 1). In contrast, soil water from the surrounding blanket bog is Na-Cl dominated and acidic (pH 5.3), whereas the water of the peat-bound karstic wetland is of intermediate character consistent with mixing of bog and spring waters (Fig. 3). Water isotope analysis revealed this water is meteoric in origin, and has undergone negligible evaporation (SI Fig. 4).

The transect across the largest mound revealed a distinct pattern of physical and chemical water properties from the centre of the mound towards the peatland (Fig. 3). The spring at the centre of the mound discharges slightly super-saturated water with respect to calcite and aragonite, representative of groundwater in close equilibrium with CaCO_3_ under conditions of elevated *p*CO_2_. As the water flows downslope towards the edge of the mound, it equilibrates with atmospheric *p*CO_2_ (degasses) resulting in increasing saturation indices (SI), causing precipitation of CaCO_3_. As Ca precipitates and the water mixes with acidic peat water (pH 5.3) towards the margin of the mound, saturation indices decrease markedly and waters are under-saturated with respect to calcite, aragonite, and dolomite. We also noted that dolomite saturation features a similar behavior compared to calcite and aragonite SI across the mound, but indices are twice as high towards the slopes of the mound (SI > 2) (Fig. 3). However, Raman spectroscopy of the precipitates indicate calcite, not dolomite, in line with the ‘dolomite problem’^31^ (that is, whereas basic chemical principles appear to allow crystallization of dolomite under common earth surface conditions, this does not occur in nature and instead dolomite precipitation appears to require either geological timescales or other special conditions). Aragonite or biologically formed amorphous calcium carbonate would result in different Raman spectra compared to crystalline calcite^32^. Our findings are consistent with the observed behavior of typical tufa-depositing springs elsewhere in the world^33^, although biotic processes (e.g. microbial metabolism) may contribute at some level to the observed chemical evolution of the water flowing across the mound.

### Stromatolite Composition

**Figure 4:**
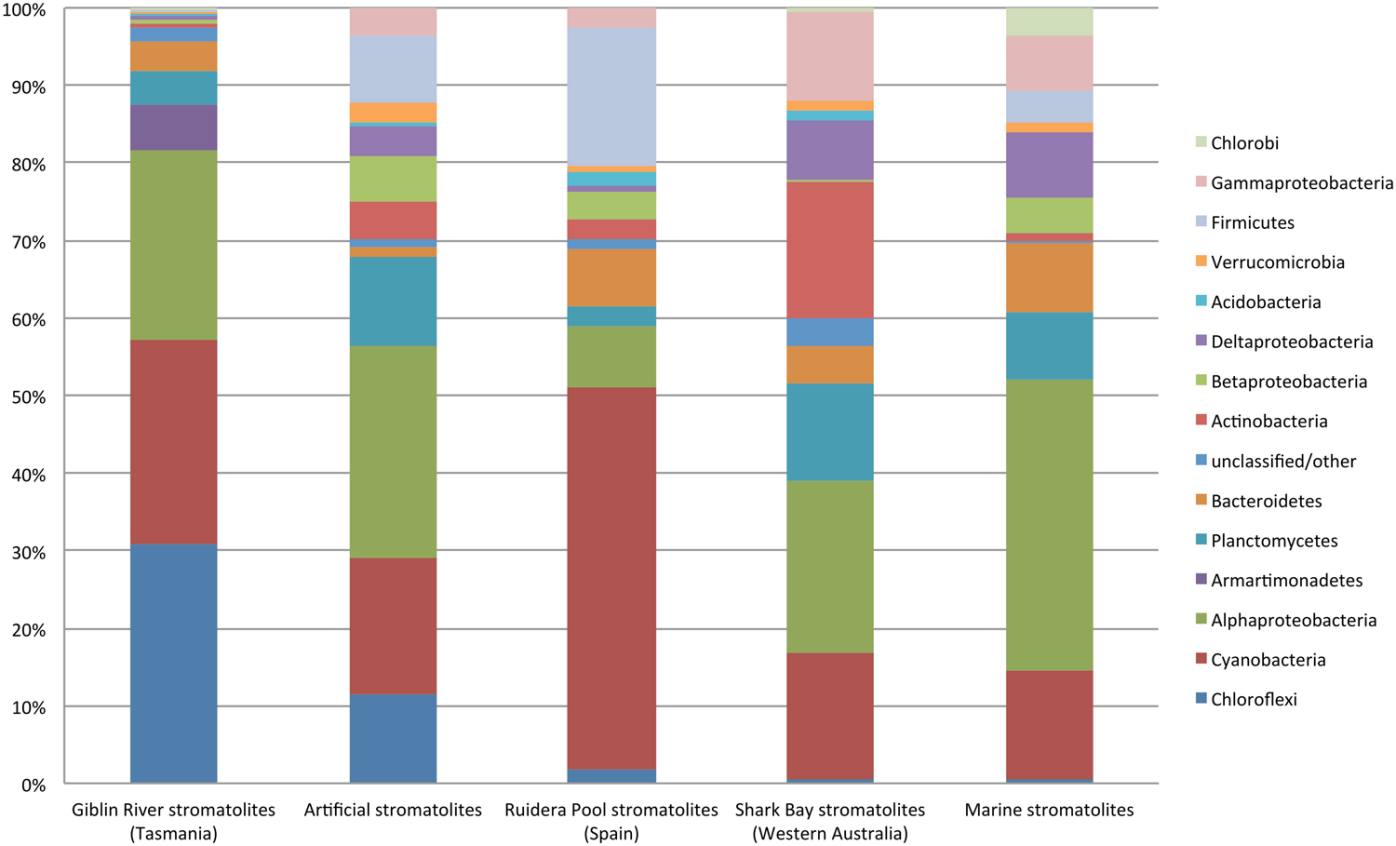
Bacterial diversity present in stromatolites defined by the proportion of 16 S rRNA gene sequences for each given taxonomic group present. The Tasmanian data is from the Giblin River (this study); artificial stromatolites derived from Type 2 Highborne Cay stromatolites^57^; freshwater stromatolites from Ruidera Pool, Spain^22^; stromatolites from Shark Bay, Western Australia^3,5,56^; marine water derived Highborne Cay stromatolites^7,10,56^; and Pavilion Lake, Canada, lake microbialites^23^.

16 S rRNA gene sequence analysis of the bacteria from two representative stromatolite samples revealed that the operational taxonomic units (OTUs) of the Giblin River stromatolite bacterial community belonged mainly to Cyanobacteria, Chloroflexi, Armatimonadetes, Alphaproteobacteria and Planctomycetes (Fig. 4). Cyanobacterial diversity (26.4% of reads) was extensive (113 OTUs) and OTUs found are largely distinct from cultured taxa, with the main phylotypes most similar to cyanobacteria from soil crust and freshwater lake and biofilms samples^34–37^. *Chloroflexi* phylotypes formed two major clades (30.8% of reads, 131 OTUs), including one that branched within class *Chloroflexia* and another that represented a branch within the class *Anaerolineae*. The latter clade was distant from the cultured representatives of the *Anaerolineaceae* ^38^, but were most similar to taxa from biofilms of rock surfaces and mineral springs such as those described by Tomcyzk-Zak *et al*.^39^ and Headd & Engel^40^. The community composition data also implied the presence of a wide diversity of heterotrophic species normally found in abundance in oligotrophic freshwater ecosystems including microbial mats and stromatolites^41,42^. These taxa include members of the genera *Caulobacter*, *Hyphomicrobium*, families Hyphomonadaceae, Sphingomondaceae, Cytophagaceae, Saprospiraceae, and the phyla Planctomycetes and Armatimonadetes. Potentially these bacteria may depend on carbon derived from cyanobacterial primary production or could assist in decomposition of the cyanobacterial mats. Overall, based on the OTUs detected, there was limited evidence of strictly anaerobic bacteria being also present in the samples, which is consistent with the samples analysed being derived from a largely active photosynthetic mat growing on tufa. Overall, the OTUs detected were generally distinct from cultured and uncultured bacterial taxa and showing no close correspondence to OTUs detected in other stromatolite systems, including freshwater stromatolites^22^ and lake microbialites^23,43^ (Fig. 4). In this respect very few of the Giblin River OTUs displayed >98% similarity to known sequences on the database, indicating a comparatively unique community. This could partly reflect a general lack of data on microbial communities associated with freshwater spring-type ecosystems.

Given the uniqueness of the microbial community composition within Giblin River spring mound stromatolite formations, they could derive from local species present in adjacent spring water, soil and rock biofilms. However, to date, none of these ecosystems have received any detailed microbiological study and thus a complete comprehension of the microbiology of the stromatolites will require analysis of the surrounding Giblin River spring and wetland system.

### Macroinvertebrates

The rapid evolution of multicellular life in the Cambrian is believed to have caused the decline of stromatolite abundance^15^. We have therefore investigated macroinvertebrate abundance at the Giblin River spring mounds. Macroinvertebrate sampling indicates that the Giblin River spring mounds host a sparse assemblage of invertebrate taxa most of which are not yet identifiable to known, described species (SI Table 2). These include a planarian (Platyhelminthes), nematodes, oligochaetes in the Naiididae and Tubificidae, and juvenile specimens of a stonefly (Plecoptera: Gripopterygidae). A small number of microcrustacea were found (Ostracoda, harpacticoid and calanoid Copepoda), but the most prominent crustaceans were specimens of *Paraleptamphopus* spp., an amphipod genus previously regarded as endemic to New Zealand^44^. Molluscan diversity is comparable with surrounding catchments^45,46^ with 7 species or forms recorded: a bivalve (*Pisidium* sp.), the Planorbidae *Glyptophysa gibbosa*, and 5 Hydrobiidae: *Phrantela singularis*, *Phrantela angulifera*, *Austropyrgus pisinnus* and 2 unidentified *Phrantela* spp., with *A. pisinnus* and *P. singularis* being most abundant. The relative abundance of other snail species is low. Many snails, both live and deceased, were affected by carbonate encrustations on the shell – in some cases the original form of dead shells were entirely obscured by bulbous masses of accreted minerals (Fig. 2E,F). The attendant increase in mass implies increased energy consumption during movement and, ultimately, the shell may become too cumbersome or heavy for the animal to maintain mobility (Fig. 2F). Even if calcification does not actually kill affected snails, it likely has a debilitating effect which increases their susceptibility to risk factors, such as disease, predation or desiccation. Ca-encrustation was observed profiling all age stages of the snails.

We hypothesize that the mortality rate is higher amongst calcified snails and that this is responsible for suppressing snail abundance on the spring mounds. This is supported by observed mass mortality of snails in the form of dense accumulations of empty shells on the slopes of the spring mounds (Fig. 2D) and sparse live snails subject to Ca-encrustation (Fig. 2E and F). It appears that snails are particularly disadvantaged on the middle and lower slopes of the spring mounds where our data indicate conditions of increasing pH and carbonate saturation, with attendant potential for mineral precipitation downslope of the mound (Fig. 3). Elevated snail mortality implies periods of reduced predation on microbial films, the food source of the aquatic snails^47^. The coexistence of macroinvertebrates and marine stromatolites has recently been reported for a site in South Africa^16^, suggesting that metazoan co-occurrence may play a reduced role in limiting stromatolite growth^48,49^. However, we observed that areas with abundant living snails, including *Phrantela*, the largest of the gastropod species that could potentially cause significant damage by grazing, did not coincide with patches of stromatolite occurrence. We therefore suggest that Ca-precipitation and encrustation of live snails regulate metazoan abundance at this site, and infer that this mechanism has contributed to the suitability of the Giblin River spring mounds as a stromatolite habitat.

## Conclusion

The discovery of stromatolites growing on spring mounds in the Giblin River catchment provides an exciting, novel example of the range of habitats known to support this ancient life form. The comparatively benign physico-chemical conditions of this habitat contrasts with the more typically extreme conditions normally associated with stromatolites. This raises the possibility that stromatolites colonize a broader range of temperate freshwater environments than presently recognized. We anticipate our findings will be a starting point for new investigations and models on modern stromatolite formation in temperate freshwater habitats.

## Methods

### Study Site

The Giblin River catchment is located in south-west Tasmania, Australia (−42°56′S, 145°45′E), and lies within the UNESCO-listed Tasmanian Wilderness World Heritage Area. This region experiences cool temperate maritime conditions dominated by ‘roaring forties’ weather off the Southern Ocean (mean annual air temperature: 13 °C; mean annual rainfall: 2512 mm; Australian Bureau of Meteorology 1981–2010 data). The Giblin River occupies a broad syncline where dissolution of Ordovician limestone has produced an undulating, low-relief surface a few tens of metres above sea level. This surface is largely covered by Tertiary siliceous alluvial gravels and Holocene blanket bogs, a form of ombotrophic peatland which mantles large tracts of western Tasmania. The vegetation is dominated by grassy to shrubby moorland, generally considered a successional disclimax related to earlier indigenous burning. The catchment is isolated from settled areas to the east (>100 km) by rugged topography and its natural values are poorly documented.

Within the Giblin River catchment occur peat-bound karstic wetlands that are regionally distinctive due to the presence of prominent spring mounds (Fig. 1). The mounds are up to 60 m in diameter with densely vegetated marshy tops, but only rise up to approximately 0.5 m above surrounding wetlands. This study site was visited twice by helicopter, on 4 December 2015 and 18 August 2016.

### Raman spectroscopy

A cross-section of a representative stromatolite sample collected near site GR2 during the first visit (December 2015, Fig. 2B) was examined by Raman point analyses at the Central Science Laboratory, University of Tasmania. The Raman spectrum was recorded on a Renishaw inVia Raman spectrometer with StreamlineHR using a laser diode excitation at 785 nm with a power output of 55 mW at the sample, 20x (NA 0.40) objective, 10 s exposure and a grating of 1200 l/mm resulting in a spectral resolution of about 1.2 cm^−1^ between 220 and 1200 cm^−1^. The spectrum was baseline corrected to remove the background fluorescence.

### Water sampling and analyses

Water sampling was undertaken at four locations within the wetland (GR1 to GR4) in December 2015 and at two locations in August 2016 (GR5 and GR6, SI Fig. 1). GR1, GR3 and GR4 are samples from within the wetland, GR2 and GR5 at locations where water flow was visible and likely to present spring water outflow. Sample GR6 was taken well within the peatland underlain by Tertiary siliceous gravel surrounding the site. During the second visit in August 2016, we also conducted water sampling along a 61.7 m long transect through the largest mound (Fig. 1, and near GR5 in SI Fig. 1). Electrical conductivity (EC), pH, temperature, and dissolved oxygen (DO) were measured *in situ* using a HACH multiprobe (HQ40d), and salinity using a salinity refractometer. Samples for ion chemistry were collected in 50 ml centrifuge tubes after 0.45 µm filtration. Samples for bicarbonate titrations were not filtered. All water samples were stored below 4 °C until further analyses. Major anions and cations were analysed by ion chromatography (Dionex ICS-3000) at the Australian Centre for Research on Separation Science (ACROSS), University of Tasmania. Bicarbonate and total alkalinity were determined by titration with HCl. Water samples were also analysed for water isotopes (δ^18^O and δ^2^H) using a Picarro Laser Cavity Ringdown Spectrometer at the Australian Antarctic Division, Hobart.

Saturation indices (SI) were calculated using the software Geochemist’s Workbench® and are defined as SI = logIAP/K, where IAP is the ion activation product and K the equilibrium constant.

### Stromatolite sampling, sequence analysis, and community composition

Two microbial samples of stromatolitic smooth mats were collected in 50 ml centrifuge tubes near site GR2 during the first visit (December 2015), and stored below 4 °C until further analysis. DNA extraction was performed on both samples using the DNeasy PowerSoil kit (Qiagen, Carlsbad, CA, USA) and 16 S rRNA gene sequence analyses were subsequently performed using services and facilities at the Australian Genome Research Facility (AGRF). PCR primers used were for the V1 to V3 domain regions of the 16 S rRNA gene including 27 F (AGA GTT TGA TCM TGG CTC AG) and 519 R (GWA TTA CCG CGG CKG CTG). Following purification of the amplicons dual indices and adapter primers were added to the amplicons using the Nextera XT Library Prep kit (Illumina, San Diego, CA). Following denaturation and pooling the libraries were sequenced using the Illumina MiSeq platform according to standard protocols generating 300 bp pairended reads. Sequence processing and assessment was performed by joining FastQ files using FastX (http://hannonlab.cshl.edu/fastx_toolkit/index.html). Reads were then quality filtered and denoised in Mothur v. 1.35.1^50,51^. Sequences with a mean PHRED score of >30 and containing bases of scores <8 were discarded. Alignment, clustering and chimera removal was then performed using UPARSE^52^. After manual inspection there was still potential evidence of chimeras amongst OTUs despite use of UCLUST as a result clusters were further checked for chimeras using DECIPHER in short sequence mode^53^. This approach was useful in flagging potential chimeras for assessment. Singleton reads were excluded from further analysis. The remaining OTUs were classified using PyNAST 1.1^54^ against the Greengenes database (March 2013 version)^55^. From the two samples 152145 and 127009 reads were obtained after filtration and grouped collectively into 1063 OTUs at the 97% level. Both samples were highly similar sharing 61.4% of the OTUs. Of the OTUs 208 were designated unclassified. Manual alignments were performed to assess their phylogenetic placement by utilizing a combination of Greengenes database and a custom NCBI 16 S rRNA gene sequence database. Of these OTUs 21 were found to be chimeric and thus culled. This polished dataset was then used to compare the taxonomic makeup with other stromatolite data available in the NCBI database^3,5,7,10,22,23,56,57^.

### Macroinvertebrate sampling

Invertebrate sampling was conducted in August 2016. Six samples were taken at points 0–5 m from the tape along the transect line. An additional three opportunistic samples targeting the surrounding streams and a dense patch of live and dead snails were also taken. Three sampling methods (core, sweep and hand collection) were applied, depending on the substrate conditions. Core sampling consisted of applying a 50 ml syringe (top removed to form a cylinder) to the substrate within a 20 cm^2^ quadrat to extract 150 ml of sandy substrate; the contents were then immediately placed into a jar and preserved in 70% ethanol. Sweep netting was used in wetter areas where core samples could not be retained. An aquarium net of 15 × 10 cm and 300 µm mesh size was used to collect substrate samples by placing the net downstream of an area 40 × 40 cm disturbed by hand to a depth of 1 cm. Finally, the hand collection method was used to collect a cross section of observable snails at various locations across the mound.

Samples were all preserved and sorted to family (or genus) under a dissection microscope. Mollusca were further identified to species using microscopy and dissection, current keys and descriptions^45,46^.

### Data availability

Representative OTU sequence data were deposited in the NCBI GenBank database under accession numbers KX903306-KX904341.

## Electronic supplementary material

Supplementary Information
