# Supplementary material for: Stromatolites on the rise in peat-bound karstic wetlands
Source: Sci Rep. 2017 Nov 13;7:15384. doi: 10.1038/s41598-017-15507-1 (PMC5684344; doi:10.1038/s41598-017-15507-1)
Supplement: Supplementary file 1 — Supplementary Information [file 41598_2017_15507_MOESM1_ESM.docx]

Stromatolites on the rise in peat-bound karstic wetlands

Bernadette C. Proemse^1, 2^, Rolan S. Eberhard^3*^, Chris Sharples^4^, John P. Bowman^5^, Karen Richards^3^, Michael Comfort^3^, Leon A. Barmuta^1^

^1^ School of Biological Sciences, University of Tasmania, Private Bag 55, Hobart, Tasmania 7001, Australia.

^2^ Australian Centre for Research on Separation Science, University of Tasmania, Tasmania 7001, Australia

^3^ Department of Primary Industries, Parks, Water & Environment, GPO Box 44, Hobart, Tasmania, 7001, Australia.

^4^ Geography and Spatial Science, University of Tasmania, Private Bag 76, Hobart, Tasmania 7001, Australia.

^5^ Tasmanian Institute of Agriculture, University of Tasmania, Private Bag 98, Hobart, Tasmania, 7001, Australia.

*corresponding author: Rolan.Eberhard@dpipwe.tas.gov.au

**Supplementary Information**


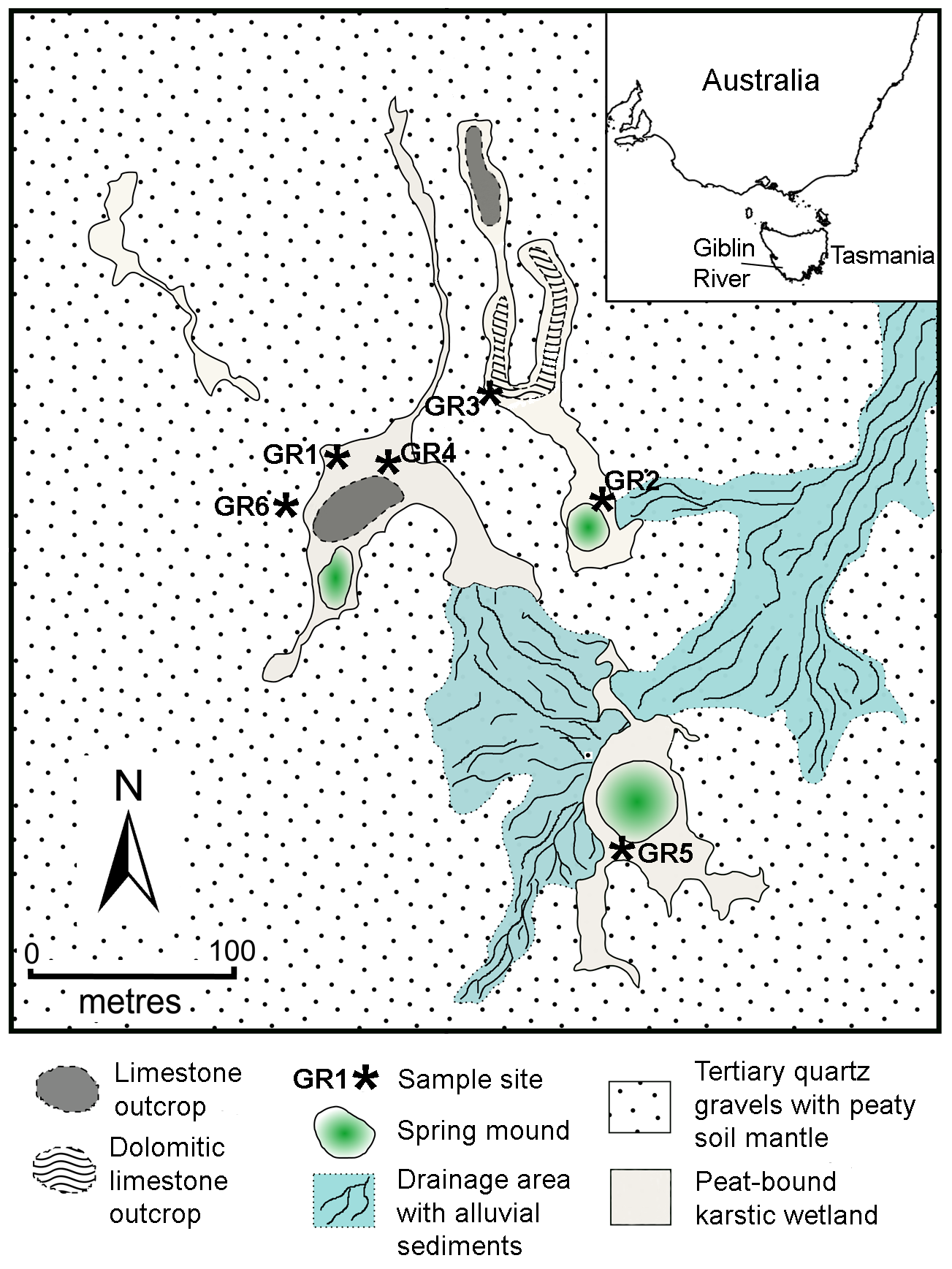


SI Figure 1: Location map and plan of the Giblin River study site, south-west Tasmania (-42°56’ S, 145°45’ E), based on ortho-rectified satellite imagery (map prepared using Google Earth satellite imagery © 2017 CNES/Airbus, Digital Globe) and field observations. The sample sites indicate water samples taken from the spring mounds (GR2, GR5), peat-bound karstic wetland (GR1, GR3, GR4) and peatland on Tertiary quartz gravel (GR6). Stromatolitic smooth mats were observed on all three spring mounds. Stromatolite material analysed in this study was collected near GR2. The red dashed line indicates the transect for water sampling across the largest mound near GR5. The map was created using Inkscape (V 0.48, www.inkscape.org) and Photoshop Elements (V 5.0, www.adobe.com/products/photoshop-elements.html).


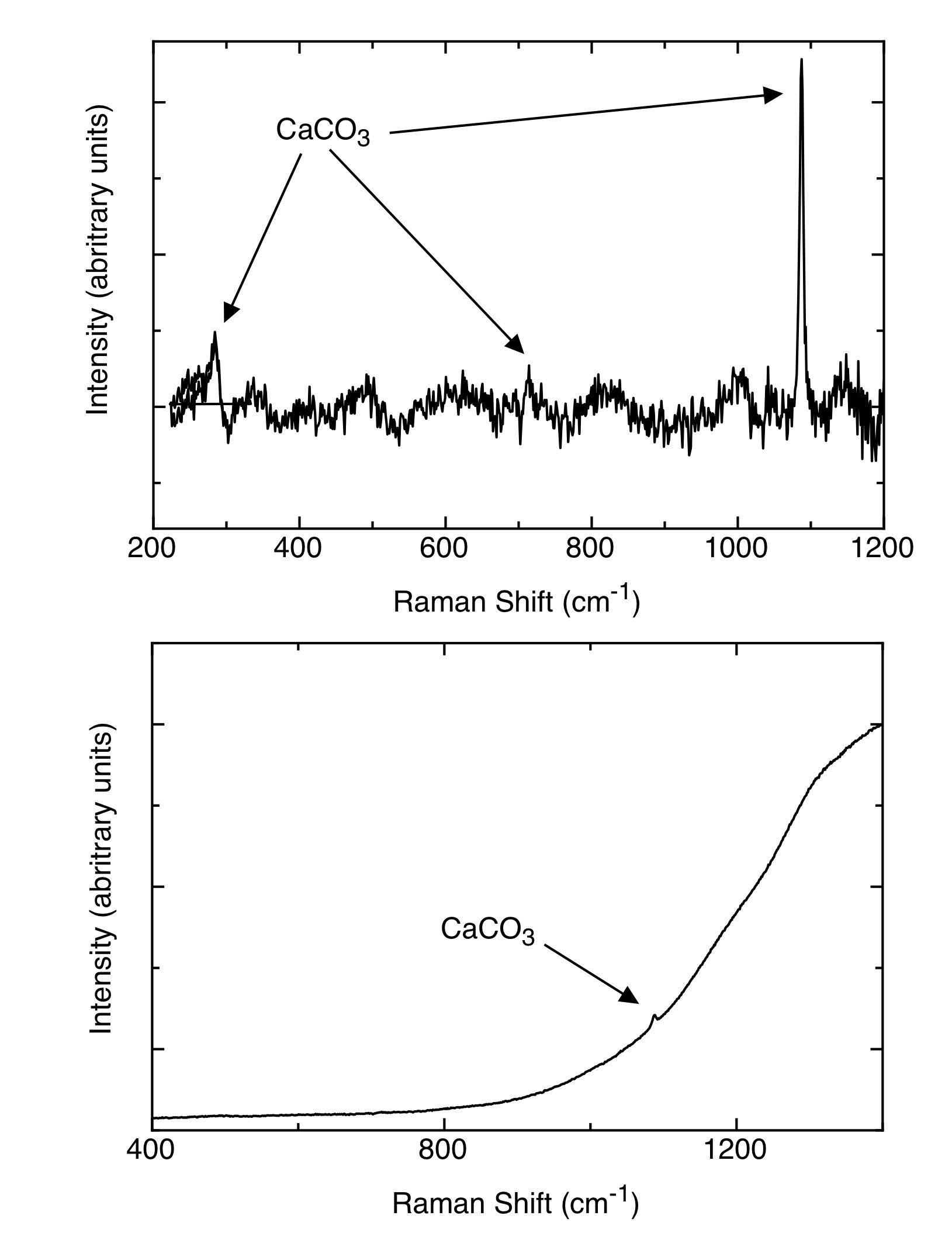


**SI Figure 2:** Raman analysis of the pale stromatolite layers reveal a distinct Raman spectrum with peaks at around 288, 719 and 1090 cm^-1^, indicative of crystalline calcite (CaCO_3_). Biologically formed amorphous calcium carbonate or aragonite would result in different Raman spectra^1^.


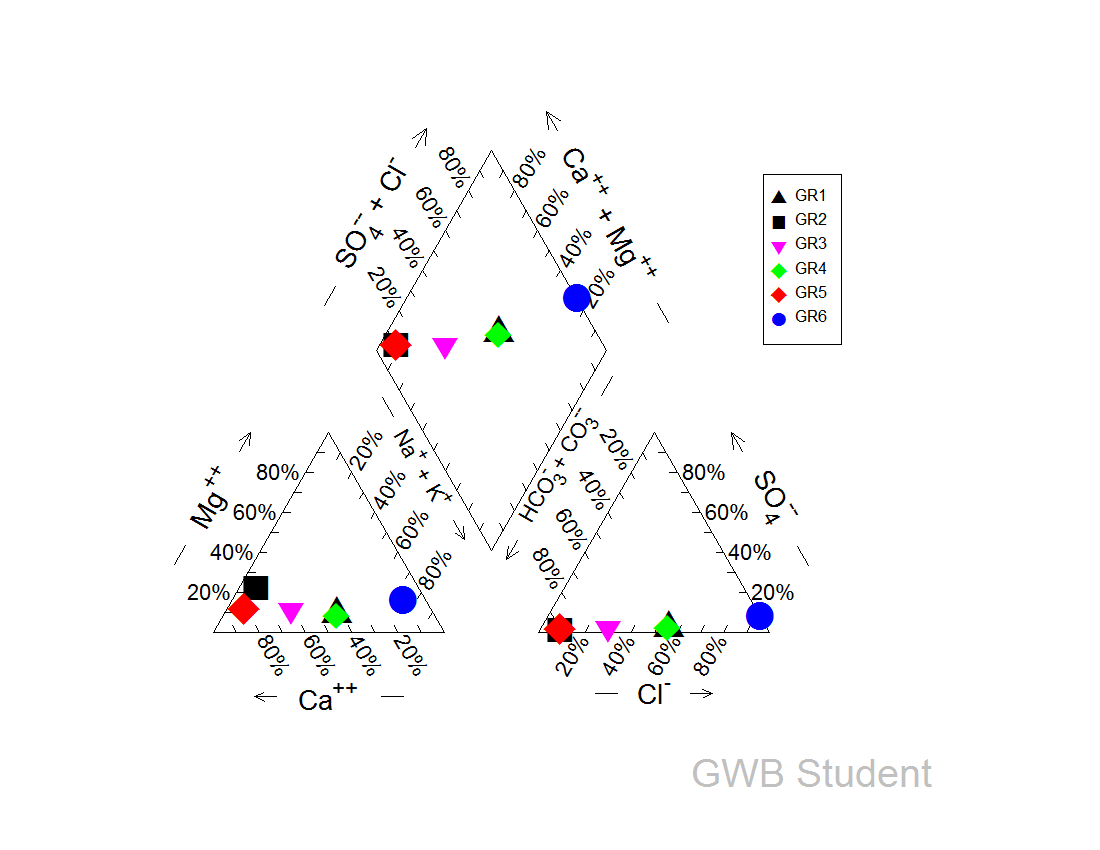


**SI Figure 3**: Piper-diagram of water samples from the Giblin River study site. The spring mound samples (GR2 and GR5) are Ca-HCO_3_ dominated, whereas the peat water (GR6) is Na-Cl dominated. Samples from the peat-bound karstic wetland (GR1, GR3 and GR4) are mixed spring and peat water.


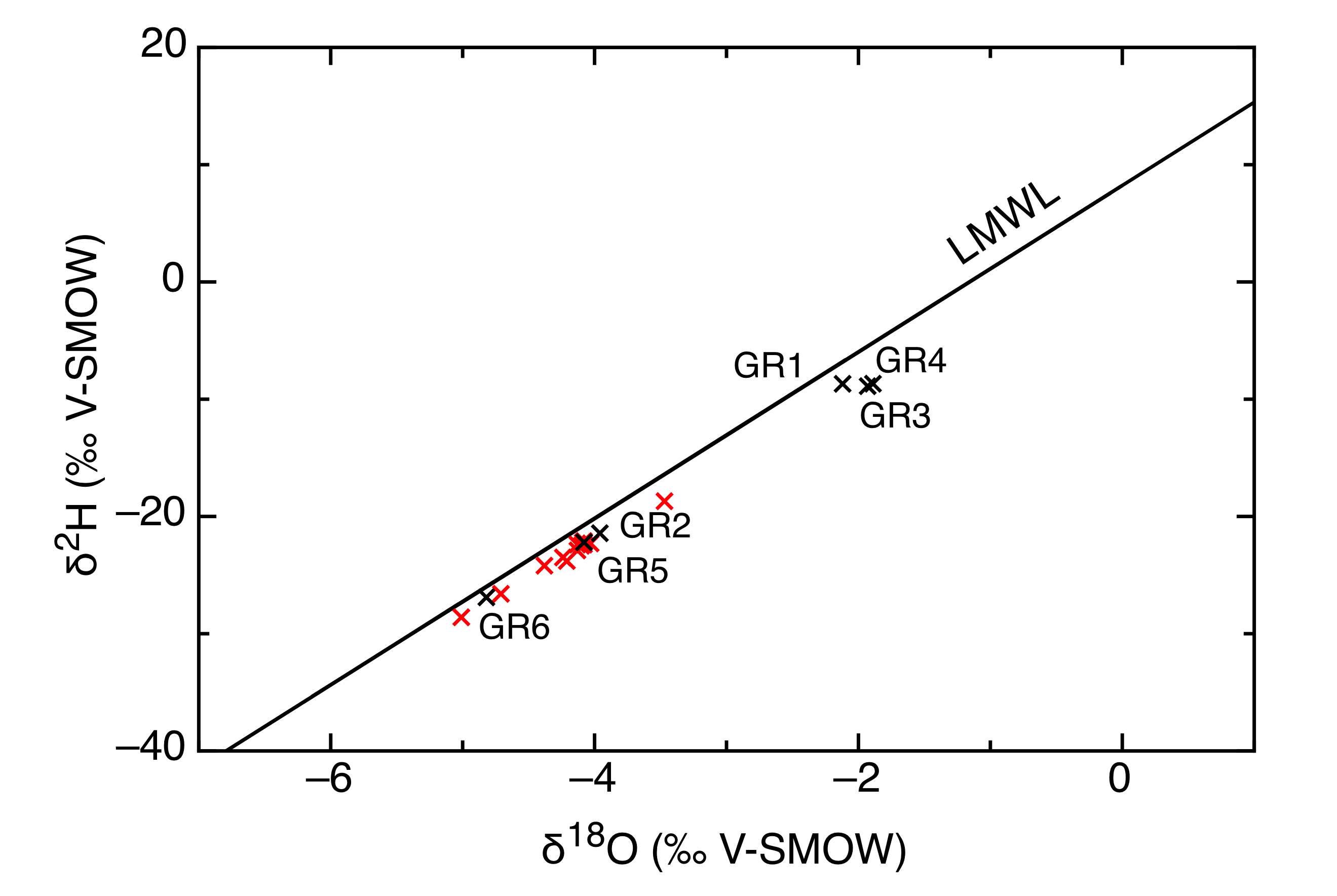


**SI Figure 4**: Water isotopic composition (δ^18^O, δ^2^H) of water samples from the Giblin River study site. Samples GR1 to GR5 were taken from the spring mounds and peat-bound karstic wetland, whereas GR6 was collected from water ponded on the surrounding peatland. Samples GR1 to GR4 samples were collected in summer 2015, GR5 and GR6 in winter 2016. Water samples collected along the mound transect near GR5 are marked in red. All samples plot close to the Local Meteoric Water Line (LMWL) reported for Cape Grim, Tasmania^2^.

**SI Table 1:** Ion chromatography results and field parameters for water samples from the Giblin River study site (see SI Fig. 1 for sample locations). Saturation indices (SI) were calculated at 25 °C using the software Geochemist’s Workbench® and are defined as SI=logIAP/K, where IAP is the ion activation product and K the equilibrium constant^3^.

| **Parameter** | **GR1**  **PKW*** | **GR2**  **spring** | **GR3**  **PKW*** | **GR4**  **PKW*** | **GR5**  **spring** | **GR6**  **peat** |
| --- | --- | --- | --- | --- | --- | --- |
| EC (µS/cm) | 107.7 | 640.0 | 187.1 | 109.3 | 618.0 | 82.2 |
| DO (mg/L) | 8.91 | 3.44 | 8.8 | 8.9 | 7.5 | 10.8 |
| DO (%) | 64.5 | 33.6 | 96.1 | 100.5 | 68.4 | 102.6 |
| pH | 6.1 | 7.6 | 7.5 | 7.1 | 7.5 | 5.3 |
| HCO_3_^-^(mg/L) | 25 | 345 | 74 | 25 | 382.0 | 0 |
| Na^+^(mg/L) | 10.5 | 11.3 | 11.7 | 10.9 | 11.5 | 8.4 |
| K^+^(mg/L) | 0.5 | 0.3 | 0.4 | 0.4 | 0.7 | 0.5 |
| Mg^2+^(mg/L) | 1.1 | 17.9 | 2.4 | 0.9 | 9.7 | 1.0 |
| Ca^2+^(mg/L) | 8.1 | 93.7 | 22.1 | 8.4 | 113.7 | 1.0 |
| Cl^-^(mg/L) | 18.9 | 19.6 | 18.2 | 18.3 | 20.3 | 16.6 |
| SO_4_^2-^(mg/L) | 1.0 | 3.0 | 1.7 | 1.0 | 5.3 | 2.0 |
| SI aragonite | -3.4 | 0.6 | -0.7 | -2.0 | 0.6 | -11.1 |
| SI calcite | -3.2 | 0.7 | -0.6 | -1.9 | 0.7 | -11.0 |
| SI dolomite | -6.2 | 1.9 | -1.0 | -3.6 | 1.6 | -20.8 |
| SI gypsum | -4.4 | -3.2 | -3.8 | -4.4 | -2.9 | -5.0 |

*PKW = peat-bound karstic wetland

.

SI Table 2: Macroinvertebrate sampling results from sites near the largest mound (GR5, SI Fig. 1).

| **collection method** | **sample number** | **location description** | Acarina | Amphipoda | Cladocera | Coleoptera | Parastacidae | Mollusca | Mollusca | Mollusca | Mollusca | Nematoda | Diptera | Diptera | Diptera | Diptera | Diptera | Odonata | Platyhelminthes | Plecoptera | Trichoptera |
| --- | --- | --- | --- | --- | --- | --- | --- | --- | --- | --- | --- | --- | --- | --- | --- | --- | --- | --- | --- | --- | --- |
|  |  |  | Anisitsiellidae sp. | Paraleptamphopus spp. |  | Dytiscidae spp. | Ombrastacoides | Hydrobiidae (Phrantela spp.) | Hydrobiidae (Austropyrgus spp.) | Bivalvia | Planorbidae |  | Ceratopogonidae | Chironomidae (Tanytarsus sp) | Simulidae | Tabanidae | Tipulidae | Gomphidae | Planaria | Gripopterygidae | Calocidae sp. |
| sweep net sample | Sample 1 | near start of transect, in inflowing stream circling mound, 2 m from transect line on western side |  | * | * |  | * | * | * | * | * |  |  | * |  | * | * | * | * | * |  |
| core sample | Sample 2 | 5 m from start, 2 m west of transect line |  | * |  |  |  | * | * | * |  | * |  | * |  |  |  |  |  |  |  |
| core sample | Sample 3 | approximately 25 m along the transect, 2 m west of the line |  | * | * | * |  | * | * |  |  |  | * |  |  |  |  |  |  |  |  |
| core sample | Sample 4 | 15 m from transect end (3 x 50 ml core) |  |  |  |  |  | * |  |  |  |  |  |  |  |  |  |  |  |  |  |
| core and opportunistic | Sample 5 | 5 m from end of transect, 2 m west of line |  |  |  |  |  | * |  |  |  |  |  |  |  |  |  |  |  |  |  |
| sweep net sample | Sample 6 | outflow stream ~ 2 m from end of transect line (in transect) |  |  |  |  |  | * | * |  |  |  |  |  |  |  |  |  |  |  |  |
| core sample | Opportunistic sample 1 | 30 m E of transect line in large accumulation of Phrantela on surface | * |  |  | * |  | *** |  |  |  |  |  |  |  |  |  |  |  |  |  |
| hand collection | Opportunistic sample 2 | 10 m E of transect line near start of transect |  |  |  |  |  | * |  |  |  |  |  |  |  |  |  |  |  |  |  |
| sweep net sample | Opportunistic sample 3 | western stream |  | * | * |  | * | * |  |  |  |  | * |  | * |  |  |  |  |  | * |

**References**

1. Weiner, S., Levi-Kalisman, Y., Raz, S. & Addadi, L. Biologically formed amorphous calcium carbonate. Connect. Tissue Res. **44**, 214-218 (2003).

2. Liu, J. *et al.* Stable isotopic compositions in Australian precipitation. *J. Geophys. Res.* **115,** D23307 (2010).

3. Bethke, C. M. Geochemical and biogeochemical reaction modeling (ed. Bethke, C.M.) (Cambridge University Press, 2008).
